# A Human iPSC Line Carrying a *de novo* Pathogenic *FUS* Mutation Identified in a Patient With Juvenile ALS Differentiated Into Motor Neurons With Pathological Characteristics

**DOI:** 10.3389/fncel.2020.00273

**Published:** 2020-09-08

**Authors:** Li Chen, Yali Wang, Jie Xie

**Affiliations:** ^1^Department of Neurology, The First Affiliated Hospital of Zhengzhou University, Zhengzhou, China; ^2^Department of Neurology, Affiliated Suzhou Hospital of Nanjing Medical University, Suzhou, China; ^3^Help Stem Cell Innovations, Nanjing Life Science and Technology Innovation Park, Nanjing, China

**Keywords:** juvenile amyotrophic lateral sclerosis, induced pluripotent stem cells, fused in sarcoma, *de novo* mutation, motor neurons

## Abstract

Human-induced pluripotent stem cells (hiPSCs) are used to establish patient-specific cell lines and are ideal models to mirror the pathological features of diseases and investigate their underlying mechanisms *in vitro*, especially for rare genic diseases. Here, a *de novo* mutation c.1509dupA (p.R503fs) in fused in sarcoma (*FUS*) was detected in a patient with sporadic juvenile amyotrophic lateral sclerosis (JALS). JALS is a rare and severe form of ALS with unclear pathogenesis and no effective cure. An induced pluripotent stem cell (iPSC) line carrying the *de novo* mutation was established, and it represents a good tool to study JALS pathogenesis and gene therapy strategies for the treatment of this condition. The established human iPSC line carrying the *de novo* *FUS* mutation strongly expressed pluripotency markers and could be differentiated into three embryonic germ layers with no gross chromosomal aberrations. Furthermore, the iPSCs could be successfully differentiated into motor neurons exhibiting the pathological characteristics of ALS. Our results indicate that this line may be useful for uncovering the pathogenesis of sporadic JALS and screen for drugs to treat this disorder.

## Introduction

Amyotrophic lateral sclerosis (ALS) is a neurodegenerative disorder characterized by progressive loss of upper and lower motor neurons, resulting in paralysis and death within an average of 3–5 years from disease onset (Fujimori et al., 2018). The mechanisms underlying the pathogenesis of ALS and effective therapeutic strategies targeting this disorder remain unclear and controversial. Juvenile amyotrophic lateral sclerosis (JALS) typically refers to disease onset before 25 years of age, and it is a rare and severe type of classical ALS. Gene mutations are the leading cause of ALS; however, the gene mutation profiles of classical ALS and JALS are different. For instance, mutations in *SOD1, TDP-43, C9ORF72, FUS, ANG, OPTN, UBQLN2*, and* ATXN2* have recently been identified in classical ALS, while mutations in *FUS, SIGMAR1*, *SPG11, ALS2, SOD1, C19ORF12, DDHD1, SETX*, and *TARDBP* were detected in patients with JALS (Avemaria et al., 2011; Daoud et al., 2012; Siddiqi et al., 2014; Wu and Fan, 2016; Liu et al., 2017; Ma et al., 2018; Naumann et al., 2019). Whereas 90% of classical ALS cases are sporadic, the proportion falls to approximately 60% for JALS (Zou et al., 2016; Chen et al., 2020). Among the causative genes most commonly mutated in ALS, the variation in the proportion of fused in sarcoma (*FUS*) mutations in classic ALS and JALS is particularly striking. For instance, *FUS* is associated with approximately 3% of familial ALS and 1% of sporadic ALS cases (Guerrero et al., 2016), but with more than 30% of JALS cases (Mackenzie et al., 2011; Zou et al., 2016). We have also previously reported that *FUS* may account for an even greater proportion of cases of sporadic JALS, perhaps as much as 90%.

Juvenile onset and very rapid progression are the main clinical characteristics of sporadic JALS, and the average survival time after onset is only approximately 1.5 years (Zufiria et al., 2016). However, the pathogenesis of sporadic JALS is unclear owing to the difficulty in collecting nerve tissue samples and the very short survival time of patients. The extensive application of induced pluripotent stem cell (iPSC) technology will promote the investigation of sporadic JALS. *FUS*, the most frequently mutated gene related to the pathogenesis of sporadic JALS, consists of 15 exons and encodes a multi-domain, dosage-sensitive protein that is associated with a variety of neurodegenerative diseases, including ALS, frontotemporal lobar degeneration (FTLD), and polyglutamine diseases. Approximately 70 different mutations have been reported in *FUS*, and most of the ALS-causative mutations occur in exons 14 and 15 (Chen et al., 2019). Missense mutations are the most commonly observed mutations, but frameshift or nonsense mutations have also been reported (Kim et al., 2015).

We have previously described a novel heterozygous mutation in exon 14 of the *FUS* gene, c.1509dupA (p.R503fs), chromosome 16, of a 17-year-old girl who had been diagnosed with ALS based on the clinical manifestations, and who had subsequently died of respiratory failure 15 months after onset (Chen et al., 2020). No family history or cognitive impairment was found for the teenager, which hinted that this was a case of sporadic JALS. The *de novo* *FUS* mutation and the rapid progression of sporadic JALS in this patient caught our attention. Therefore, after obtaining informed consent from the patient and her family members, and after having obtained ethical approval, we decided to reprogram the patient’s peripheral blood mononuclear cells (PMBCs) into an iPSC line aiming to provide a cell model for future research on the pathogenesis of JALS and exploration of an effective treatment against this disorder. This was completed under protocol number 2020-KY-098, released by the Ethical Committee of the First Affiliated Hospital of Zhengzhou University.

## Materials and Methods

### Cell Culture and iPSC Reprogramming

iPSC lines were generated from PBMCs of the patient. PBMCs were isolated from the peripheral blood of the patient by centrifugation using a Vacutainer^®^ CPT™ (BD Biosciences, San Jose, CA, USA). After 3–4 days of culture, the PBMCs were transduced with Sendai virus using the CytoTune™-iPS 2.0 Sendai Reprogramming Kit (Thermo Fisher Scientific, Waltham, MA, USA) for 48 h. At day 3 post transduction, the Sendai virus was removed and cells were replated onto 96-well culture plates. Monoclones with dense cell growth formed after continuous culture for approximately 30 days. Healthy clones were selected and replated onto 24-well plates coated with Matrigel (Corning, NY, USA) and cultured in mTeSR1 medium (STEMCELL Technologies, BC, Canada). The mTeSR1 medium was changed every day. When the cell density had reached 70–80% confluence, ACCUTASE (Life Technologies, MD, USA) was used for normal digestion and passaging at a density of 1:4–1:8. During subculture, the ROCK inhibitor y-27632 dihydrochloride (Tocris, Bristol, UK) was added into the medium to promote iPSC adherence and a single-cell layer was formed to cover the surface of the culture vessel. Cells were maintained at 37°C in a humidified atmosphere with 5% CO_2_. Pluripotency was assessed by immunofluorescence for OCT4 and flow cytometry for SSEA-4, TRA-1-60, and OCT4.

### Flow Cytometry

Cells were detached with ACCUTASE. The fixation/permeabilization procedure was performed using the BD Cytofix/Cytoperm Kit (BD Pharmingen™, CA, USA) according to the manufacturer’s instructions. Cells were incubated at room temperature for 1 h. The percentage of PSCs was determined by SSEA-4 and TRA-1-60 antibody staining (diluted in 1% BSA), followed by flow cytometric analysis (Beckman Coulter CytoFLEX, FL, USA; software: CytExpert).

### Immunofluorescence Assay

Cells (undifferentiated hiPSCs and trilineage-differentiated cultures) were fixed in 4% PFA for 15 min, permeabilized with Dulbecco’s phosphate-buffered saline (DPBS) containing 1% BSA and 0.1% Triton X-100 for 15 min, and blocked in blocking solution (0.1% Triton X-100 and 10% FBS in PBS) for 1 h at room temperature. Samples were then incubated overnight at 4°C with primary antibodies (Supplementary Table S1), and then with the appropriate secondary antibodies (Supplementary Table S1). Hoechst (Thermo Fisher Scientific, Waltham, MA, USA) was used to stain the nuclei. Images were acquired with an inverted fluorescence microscope (Axio Observer, Zeiss, Jena, Germany).

### *In vitro* Trilineage Differentiation of hiPSCs

At passage 40, hiPSCs were harvested and differentiated into separate lineages. Trilineage differentiation was performed using the STEMdiff™ Trilineage Differentiation Kit (STEMCELL Technologies, BC, Canada) according to the manufacturer’s indications. After 5–7 days, cells were fixed in 4% PFA for immunofluorescence staining against specific ectodermal, mesodermal, and endodermal markers.

### Motor Neuron Precursor (MNP) Specification and MN Differentiation

To generate MNPs, hiPSCs were first dissociated with dispase (1 mg/ml^−1^) and split 1:6 on Matrigel-coated plates. The following day, the PSC medium was replaced with a chemically defined neural medium consisting of DMEM/F12, neurobasal medium at 1:1, 0.5× N2, 0.5× B27, 0.1 mM ascorbic acid (Santa Cruz Technologies, Santa Cruz, CA, USA), 1× Glutamax, and 1× penicillin/streptomycin (Invitrogen, Carlsbad, CA, USA). CHIR99021 (3 mM, Torcris, Bristol, UK), 2 mM DMH1 (Torcris, Bristol, UK), and 2 mM SB431542 (Stemgent, Cambridge, MA, USA) were added to the medium. hiPSCs were maintained under this condition for 6 days. The culture medium was changed every other day. Then, as a second step, the cells were first dissociated with dispase (1 mg/ml^−1^) and split 1:6 with the above-described medium. Retinoic acid (RA; 0.1 mM, Stemgent) and 0.5 mM purmorphamine (Stemgent, MA, USA) were added in combination with 1 mM CHIR99021, 2 mM DMH1, and 2 mM SB431542. The medium was changed every other day. The cells maintained under this condition for 6 days differentiated into *OLIG2^+^* MNPs.

The *OLIG2^+^* MNPs were expanded with the medium containing 3 mM CHIR99021, 2 mM DMH1, 2 mM SB431542, 0.1 mM RA, 0.5 mM purmorphamine, and 0.5 mM valproic acid (VPA; Stemgent, Cambridge, MA, USA), and split 1:6 once a week with dispase (1 mg/ml^−1^) for 2 weeks. Some of the MNPs were frozen in regular freezing medium (DMEM/F12, 10% fetal bovine serum, and 10% DMSO) in liquid nitrogen while the remainder were used for differentiation into MNs.

To induce MN differentiation, *OLIG2^+^* MNPs were first dissociated with dispase (1 mg/ml^−1^) and cultured in suspension for 6 days in the above-described neural medium supplemented with 0.5 mM RA and 0.1 mM purmorphamine. The medium was changed every other day. These cells were then dissociated with Accumax (eBioscience Inc., San Diego, CA, USA) into single cells, plated on Matrigel-coated plates, and then cultured with 0.5 mM RA, 0.1 mM purmorphamine, and 0.1 mM compound E (Calbiochem, DA, Germany) for 10 days to mature into MNs.

### Karyotyping

Briefly, hiPSCs from passage 10 were treated with 10 μg/ml colcemid (Gibco, NY, USA) for 60 min at 37°C. Subsequently, the cells were dissociated with Accutase, treated with a 0.075 M hypotonic KCl solution, and then fixed in Carnoy’s fixative. Five metaphase spreads were prepared and examined by G-banding analysis. Karyotyping was performed at the 450-band level using Genikon software (Nikon, Italia) and described in accordance with ISCN 2016.

### Mycoplasma Test

Mycoplasma contamination was checked using the Mycoplasma Detection Kit with UDG PCR Mix and Loading Dye from ExCell Bio™ (Shanghai, China) following the manufacturer’s instructions. The results of the mycoplasma test showed that this hiPSC line ZZUNEUi010-A was mycoplasma-free.

### Sendai Virus Residue Detection

The presence of Sendai virus residue was detected by PCR and quantitative real-time PCR, as described in section RNA Isolation and Quantitative Real-Time PCR.

### Whole-Exome Sequencing

Total DNA was extracted from PBMCs of the patient, the patient’s parents, and hiPSCs. Whole-exome sequencing was performed using Illumina paired-end sequencing and Agilent SureSelect Human All Exon V6 for capture and construction, double end (paired-end) sequencing strategy (raw data >12G, Q30 ≥80%). Following the identification of the *FUS* mutation, the result was validated by Sanger sequencing. The primers (5′–3′) for sequencing the targeted mutation were FUS-ex14_F1: TCACATGGGTAAGAAAGGCAGA and FUS-ex14_R1: ACAACCTCAGGTCTTTCCACCT.

### Short Tandem Repeat (STR) Analysis

The STR analysis was carried out by Guangzhou Cellcook Biotech Co., Ltd, China. Briefly, gDNA was extracted from PBMCs of the patient and hiPSCs with the DNeasy Blood and Tissue Kit (QIAGEN, Duesseldorf, Germany). Microsatellite DNA locus amplification was carried out by PCR using the STR Multi-Amplification Kit (PowerPlex 18D System) according to the manufacturer’s instructions. The PCR products of two multiplexes (STR loci and the sex-linked gene amelogenin) were assayed with 3100 DNA Analyzer (Applied Biosystems^®^). The following loci were tested: AMEL, D3S1358, D1S1656, D6S1043, D13S317, PentaE, D16S539, D18S51, D2S1338, CSF1PO, Penta D, TH01, vWA, D21S11, D7S820, D5S818, TPOX, D8S1179, D12S391, D19S433, and FGA. The results were analyzed by ABI Genotyper software.

### RNA Isolation and Quantitative Real-Time PCR

Total RNA was isolated using RNAiso Plus (Takara, Tokyo, Japan). cDNA was synthesized from 1 μg of total RNA using the Takara PrimeScript™ RT reagent Kit with gDNA Eraser (Takara) according to the manufacturer’s instructions. Quantitative real-time PCR was performed in an Applied Biosystems 7500 real-time PCR platform using Fast SYBR Green PCR Master Mix (Applied Biosystems) containing primers for pluripotency genes. The primers (5′–3′) for Sendai virus were F: TCACTAGGTGATATCGAGC and R: ACCAGACAAGAGTTTAAGAGATATGTATC. The primers (5′–3′) for FUS were F: AATAAATTTGGTGGCCCTCGG and R: GTTGCCTCTCCCTTCAGCTT.

### Western Blot

Cells were lysized in lysis buffer (1% Triton X-100, 1% SDS, 500 mM NaCl, 1 mM EDTA, and 50 mM Tris-Cl, pH 7.4) with the protease inhibitor cocktail and were boiled in sample buffer. Samples were electrophoresed on 12% Bis-Tris polyacrylamide gels and then were transferred to a PVDF (Millipore) membrane and non-specific bindings were inhibited by 5% milk. Antibodies against FUS (ab124923, Abcam), GAPDH (AF0006, Beyotime, Shanghai, China) were diluted to 1:1,000, respectively and the membranes were incubated overnight at 4°C. After incubation for 1 h at room temperature with second antibodies, proteins were detected using a Chemiluminescence Imaging System (Bio-RAD, ChemiDoc Touch).

### Statistical Analysis

Values are expressed as mean ± SD. Statistical significance was calculated with GraphPad Prism 6 (GraphPad Software). A two-tailed non-paired *t*-test was used to compare differences between two groups. *P*-values less than 0.05 were considered significant differences.

## Results

### Resource Utility

We previously reported a *de novo* pathogenic *FUS* mutation (c.1509dupA:p.R503fs) in a patient with sporadic JALS (Chen et al., 2020). In this study, we reprogrammed PBMCs derived from the patient to generate a hiPSC line carrying the novel *FUS* mutation. The specific background information for this hiPSC line (CMF001-A; hPSCreg Name, ZZUNEUi010-A) is shown in hPSCreg^1^.

### Reprogramming of Cells From the Patient

**Figure 1:**
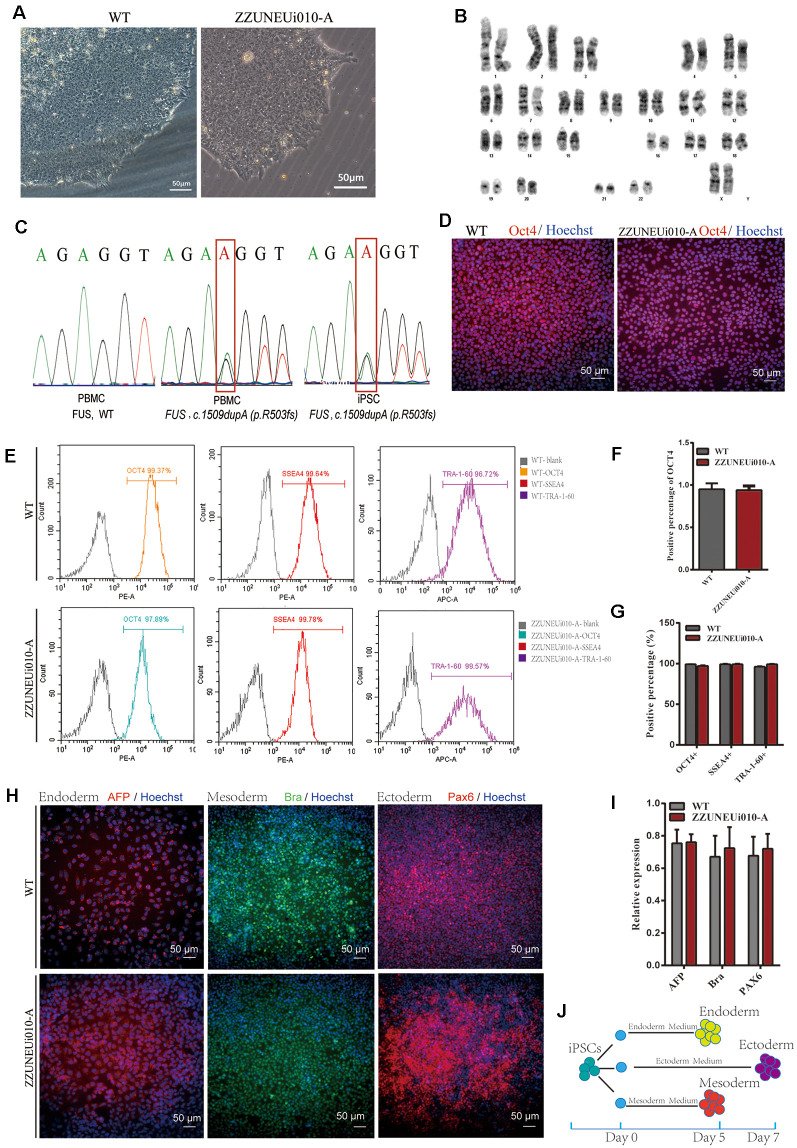
Pluripotency identification and trilineage differentiation of the human-induced pluripotent stem cells (hiPSCs). **(A)** Brightfield images of ZZUNEUi010-A clones (×10). **(B)** Karyotype analysis of ZZUNEUi010-A, 46, XX, without chromosomal aberrations. **(C)** Illumina and Sanger sequencing of PBMCs and ZZUNEUi010-A from this patient detected the same fused in sarcoma (*FUS*) mutation (c.1509dupA:p.R503fs). **(D)** Immunofluorescence images showing that ZZUNEUi010-A colonies stained strongly for the pluripotency marker OCT4. **(E)** Flow cytometry showed that the hiPSCs were strongly positive for the pluripotency markers OCT4 (97.89%), SSEA-4 (99.78%), and TRA-1-60 (99.57%). **(F)**There was no significant difference between ZZUNEUi010-A and WT in positive staining rate for OCT4 (*n* = 3 views, *p* > 0.05). **(G)** There was no significant difference between ZZUNEUi010-A and WT in positive rate for those markers OCT4, SSEA-4, and TRA-1-60 (*n* = 3, *p* > 0.05). **(H)** On day 5, the cells differentiated from the hiPSCs stained strongly for AFP and brachyury respectively, while at day 7 the differentiated cells showed strong PAX6 staining. **(I)** There was no significant difference between ZZUNEUi010-A and WT in positive staining rate for the three embryonic markers (*n* = 3 views, *p* > 0.05). **(J)** Schematic diagram of triploblastic differentiation experiment.

The hiPSC line (CMF001-A; ZZUNEUi010-A) was established by retrovirus-mediated reprogramming of PBMCs derived from a patient with sporadic JALS. We analyzed the iPSC clones by brightfield microscopy and immunofluorescence staining. After continuous culture of the reprogrammed cells for approximately 30 days, the clones proliferated and spread evenly, as can be seen in Figure 1A. G-banded karyotype analysis showed that CMF001-A had a normal 46, XX karyotype (Figure 1B). The hiPSCs were cultured in feeder-free conditions after being stably cloned. No Sendai virus residue (Supplementary Figure S1A) or mycoplasma contamination was detected (Supplementary Figure S1B). To determine whether the mutation present in the hiPSC line was the same as that in the PBMCs obtained from the patient, genomic DNA was extracted for whole-exome sequencing, followed by Sanger-sequencing verification, using the primers FUS-ex14_F1 and FUS-ex14_R1. The results showed that the same heterozygous mutation c.1509dupA (p.R503fs) was present in both the hiPSCs and PBMCs but not in the WT (PBMCs from the patient’s mother) sample (Figure 1C). The STR results for the hiPSC line also matched those of the PBMCs of the patient, indicating that the hiPSCs were patient-specific (Supplementary Figure S1C). These results suggested that the hiPSC line, derived from the patient and harboring the patient’s *de novo* mutated gene, was successfully established without contamination or mutation during culture.

### Pluripotency and Trilineage Differentiation Potential of the hiPSCs

OCT4 is a key transcription factor for cell reprogramming and functions as a positive regulator of genes required for the maintenance of embryonic stem cell (ESC) pluripotency (Karagiannis et al., 2019). Immunofluorescence staining showed that OCT4 was strongly expressed in the iPSC clones (Figure 1D). There was no significant difference between ZZUNEUi010-A and WT in positive staining rate for OCT4 (Figure 1F). At passage 10, three pluripotency markers (OCT4, SSEA-4, and TRA-1-60) were utilized to determine the pluripotency potential of the hiPSCs. The positive expression of these markers was measured by flow cytometry using human IgG as the isotype control. The expression rates of OCT4, SSEA-4, and TRA-1-60 were high, showing values of 97.89, 99.78, and 99.57% (Figure 1E), respectively, suggesting that this hiPSC line presented pluripotential potential. There was no significant difference between ZZUNEUi010-A and WT in positive rate for those markers (Figure 1G). At passage 40, the hiPSCs showed stable proliferative potential. Some of these hiPSCs were harvested and induced to differentiate into the three embryonic germ layers using the STEMdiffTM Trilineage Differentiation Kit after single-cell clone inoculation on culture plates followed by 5–7 days of culture (Figure 1J). Three embryonic markers (AFP, an endodermal marker; brachyury, a mesodermal marker; and PAX6, an ectodermal marker) were used to determine the trilineage differentiation potential of the hiPSCs. On day 5, the cells differentiated from the hiPSCs stained strongly for AFP and brachyury respectively, while at day 7 the differentiated cells showed strong PAX6 staining (Figure 1H). There was no significant difference between ZZUNEUi010-A and WT in positive staining rate for the three embryonic markers (Figure 1I).

### MNP Specification and MN Differentiation From the hiPSCs

**Figure 2:**
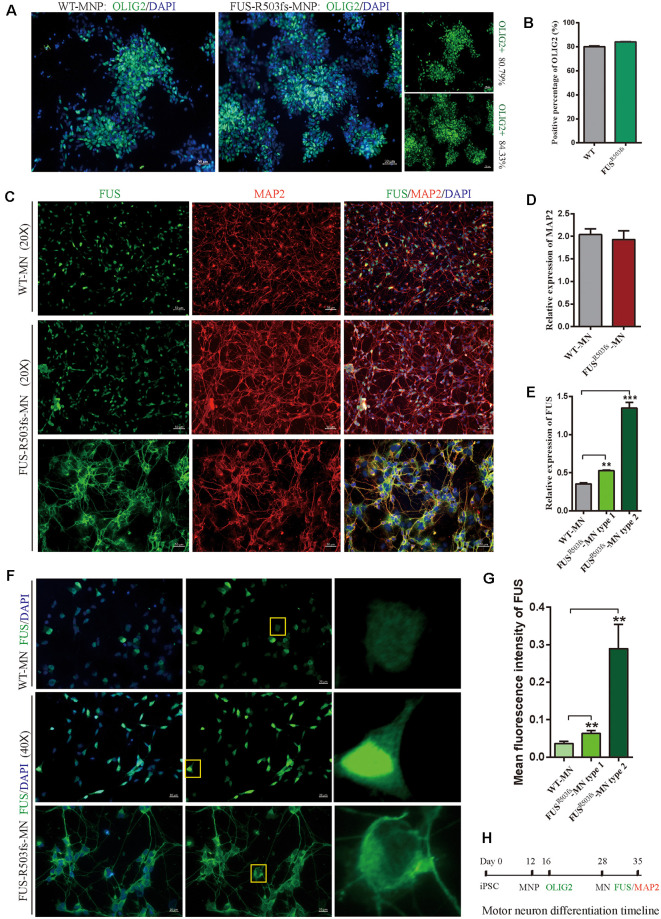
Cells from the hiPSC line (ZZUNEUi010-A) could be differentiated into motor neurons and exhibited pathological characteristics of amyotrophic lateral sclerosis (ALS). **(A)** Representative images of motor neuron precursors (MNPs) on day 16 showing strong OLIG2 (green) staining. **(B)** There was no significant difference between FUSR503fs-MNPs and WT-MNPs in positive staining rate for OLIG2 (*n* = 3, *p* > 0.05). **(C)** Representative images of motor neurons on day 35 showing positive MAP2 (red) and FUS (green) staining. **(D)** There was no significant difference between FUSR503fs-MNP and WT-MNP in positive staining rate for MAP2 (*n* = 3, *p* > 0.05). **(E)** The relative expression of FUS was significantly higher in MNs of the FUSR503fs group than in those of the WT group (*n* = 3, **p < 0.01, ***p < 0.001). **(F)** Representative images of FUS-positive staining MNs in WT, *FUS*^R503fs^–MN type 1, and *FUS*^R503fs^–MN type 2 groups. **(G)** The mean intracellular FUS fluorescence intensity of single cell was significantly higher in MNs of the *FUS*^R503fs^ group than in those of the WT group (WT-MNs, *n* = 142; *FUS*^R503fs^–MN type 1, *n* = 146; *FUS*^R503fs^–MN type 2, *n* = 75; **p < 0.01). **(H)** Schematic diagram of motor neuron differentiation process.

Twenty-eight days after the induction of differentiation, which involved two stages (MNP specification and MN differentiation) and four steps (Du et al., 2015), the iPSCs differentiated into MNs. In the first stage, hiPSCs differentiated into MNPs over 12 days. The MNPs were allowed to proliferate from day 12 to day 27, and they were identified by OLIG2 staining on day 16 (Figure 2H). The representative photos showed that the positive staining rate for OLIG2 expression was 80.79% in wild-type (WT) MNPs and 84.33% in MNPs harboring *FUS*^R503fs^ (Figure 2A). There was no significant difference between *FUS*^R503fs^-MNP and WT-MNP in positive staining rate for OLIG2 (Figure 2B). On day 28, the MNPs were placed in a special neural medium to induce differentiation into MNs. Subsequently, MNs gradually matured and could be cultured *in vitro* for long periods. After 7 days of differentiation (day 35, Figure 2H), differentiating MNs were stained for MAP2 and FUS (Figure 2C). We found that both the *FUS*^R503fs^ group and the WT group developed axons and dendrites, displayed the morphological characteristics of typical neurons, and expressed MAP2 widely. There was no significant difference between *FUS*^R503fs^-MNP and WT-MNP in positive staining rate for MAP2 (Figure 2D).

### The MNs Differentiated From the hiPSCs Showed Pathological Characteristics of JALS

**Figure 3:**
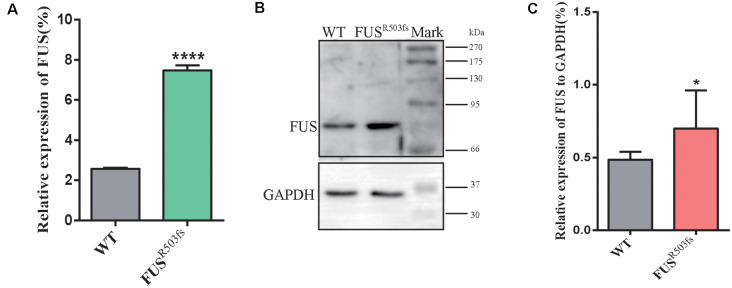
FUS expression was significantly higher in the FUS^R503fs^ than that in WT group. **(A)** The Q-rtPCR results indicated that the mRNA expression level of FUS was significantly increased in FUS^R503fs^ compared with WT group (*n* = 6, ****p < 0.0001). **(B)** Western blot analysis of FUS protein in neurons of WT and FUS^R503fs^. Molecular weight markers are shown on the right. **(C)** Quantification of FUS protein levels in FUS^R503fs^ neurons was significantly increased compared with WT group (*n* = 8, *p > 0.05).

The FUS staining showed interesting results. In the WT group, FUS-positive staining was primarily localized to the nucleus, while in *FUS*^R503fs^ group, FUS-positive staining appeared to be stronger than that in WT cells and was characterized by two types: *FUS*^R503fs^–MN type1, where FUS staining was strong in the nucleus and weak in the cytoplasm; and *FUS*^R503fs^–MN type 2, where FUS staining was weak in the nucleus and strong in the cytoplasm, cytoplasmic axons, and dendrites (Figures 2C,F). These two types of FUS distribution characteristics did not exist in the WT control group neurons. It was possibly the phenomenon of varying degrees of pathological aggregation and distribution of aberrant FUS in neurons (Scekic-Zahirovic et al., 2017). The relative expression of FUS was significantly higher in MNs of the *FUS*^R503fs^ group than in those of the WT group (Figure 2E). The mean intracellular FUS fluorescence intensity of single cell was significantly higher in MNs of the *FUS*^R503fs^ group than in those of the WT group (Figure 2G). This suggested that the *FUS* mutation-induced pathological characteristics of JALS—aberrant cytoplasmic aggregation and reduced nuclear entry of FUS—could be successfully recapitulated in hiPSC-derived MNs. In order to verify the change of FUS expression caused by this gene mutation in motor neurons, the expression of FUS in *FUS*^R503fs^ group and WT group was detected by quantitative real-time PCR (Q-rtPCR) and protein immunoimprinting (Western Blot). The expression level of FUS mRNA in *FUS*^R503fs^ group neurons was significantly higher than that in the WT group (Figure 3A). The expression level of FUS protein in *FUS*^R503fs^ group neurons was significantly higher than that in the WT group (Figures 3B,C). Moreover, these results indicated that our hiPSC line, ZZUNEUi010-A, has potential for use as a cell model to study sporadic JALS.

## Discussion

With the development of virus-free reprogramming and gene correction technologies, hiPSCs are becoming widely used in classical ALS research. However, JALS cases, especially sporadic JALS, associated with different genetic mutations have increasingly been reported in the past decade due to advances in gene sequencing technology. The hiPSCs derived from our patient with JALS will play an important role in the study of the pathogenesis of this condition and its rapid progress. As previously reported, ALS-causative *FUS* mutations are rare in adults (Guerrero et al., 2016) but more common in JALS (Mackenzie et al., 2011; Zou et al., 2016). However, why the *FUS* mutation appears so disproportionately in different age groups remains unclear. In our study, we successfully established a hiPSC line derived from a patient with sporadic JALS carrying the *de novo* *FUS* mutation c.1509dupA:p.R503fs. This hiPSC line could be differentiated into motor neurons and showed a typical cytoplasmic *FUS* aggregation, suggesting that it has good potential as a tool for use in JALS research.

FUS is a DNA/RNA-binding protein with functional homology to TDP-43 and belongs to the FET/TET protein family (Guerrero et al., 2016). It is a 526 amino acid, multidomain protein, with an N-terminal transcriptional activation domain, multiple nucleus-binding domains, and a C-terminal nuclear localization signal (NLS; Dormann and Haass, 2013). FUS shuttles continuously between the nucleus and the cytoplasm, regulating gene expression and performing numerous cytoplasmic functions (Zinszner et al., 1997; Birsa et al., 2019) such as DNA damage repair and regulation of mRNA stability, autophagy, RNA granule assembly, and ER-mitochondria associations (An et al., 2019). *FUS* levels in the cytoplasm are extremely low under physiological conditions, while aberrant FUS mislocalizes and accumulates in the cytoplasm in end-stage ALS (Kapeli et al., 2017). In our experiment, this typical pathological change was observed 7 days after the maturation of MNs. The *de novo* mutation R503fs, like most *FUS* mutations in patients with JALS, is located in the NLS domain (Loughlin and Wilce, 2019; Chen et al., 2020). The NLS domain is essential for FUS to enter the nucleus and regulate the transcription of DNA and cytoplasmic localization (Dormann and Haass, 2013; Casci et al., 2019). FUS mutation in the NLS domain may be associated with cytoplasmic aggregation of aberrant protein. The gain of toxic function in the cytoplasm and the loss of function in the nucleus due to the FUS–NLS mutation may be associated with the early onset and rapid progression of JALS (Baumer et al., 2010; Conte et al., 2012; Scekic-Zahirovic et al., 2016). However, the exact pathogenesis remains unclear, and no effective medication is currently available for the treatment of this disorder.

The difficulty in obtaining nerve tissue from patients and the lack of ideal animal models pose great challenges to the study of sporadic JALS. Models of JALS suffer from the disadvantages of late-onset and the lack of typical pathological characteristics. For instance, Devoy and colleagues created a mouse model of ALS harboring a human-associated frameshift mutation in *FUS* (p.G466VfsX14), and found progressive motor neuron degeneration in 15-month-old mice without aggregation of aberrant proteins (Devoy et al., 2017), which could be explained by the functional redundancy between FUS and other FET proteins that occurs in neurons (Kabashi et al., 2011; Sasayama et al., 2012). Therefore, patient-derived iPSCs may be a better choice as a disease model for sporadic JALS. However, only a few hiPSC lines have been established for use in investigating the pathomechanisms of ALS, including *FUS*^P525L^ (De Santis et al., 2017; Errichelli et al., 2017; Marrone et al., 2018); *FUS*^H517D^ (Ichiyanagi et al., 2016); *FUS*^R521H^(Guo et al., 2017); *FUS*^R495QfsX527^ (Naujock et al., 2016); *FUS*^G504Wfs*12^ and *FUS*^Q519E^ (Lim et al., 2016); and *FUS*^Asp502Thrfs*^ and *FUS*^R521C^ (Higelin et al., 2016). Although all of these iPSC lines can be differentiated into MNs that exhibit typical pathologic characteristics of ALS, most were derived from patients with classical ALS or familial JALS. In our study, c.1509dupA:pR503fs, a *de novo* mutation in *FUS*, was detected in a sporadic JALS case, which will be of great research value. A hiPSC line named ZZUNEUi010-A, and carrying the *FUS*^R503fs^ mutation, was subsequently generated through the reprogramming of PBMCs derived from the patient with JALS. The hiPSC showed strong expression of pluripotency markers (OCT4, SSEA-4, and TRA-1-60) and could be differentiated into three embryonic germ layers, as evidenced by the detection of AFP (endoderm), brachyury (mesoderm), and Pax6 (ectoderm) marker expression. Moreover, no gross chromosomal aberrations or specific copy number variations were observed. Importantly, we induced the differentiation of the hiPSCs into MNs that exhibited pathological characteristics of ALS. Overexpressed FUS cytoplasmic aggregation were observed in motor neurons differentiated from ZZUNEUi010-A.

In conclusion, a cell model of sporadic JALS (CMF001-A)^1^ carrying a *de novo* *FUS* mutation c.1509dupA:p.R503fs was successfully established. This cell line can be differentiated into motor neurons with pathological features of ALS and represents a good tool as well as a new strategy for exploring the pathogenesis and treatment of sporadic JALS.

## Data Availability Statement

The datasets presented in this study can be found in online repositories. The names of the repository/repositories and accession number(s) can be found in the article/Supplementary Material.

## Ethics Statement

The studies involving human participants were reviewed and approved by The ethics committee of the First Affiliated Hospital of Zhengzhou University (2020-KY-098). The patients/participants provided their written informed consent to participate in this study.

## Author Contributions

LC performed most of the experiments and wrote and submitted the manuscript. YW reviewed and revised the manuscript. JX performed the sequencing analysis.

## Conflict of Interest

The authors declare that the research was conducted in the absence of any commercial or financial relationships that could be construed as a potential conflict of interest.

## Footnotes

^1^https://hpscreg.eu/cell-line/ZZUNEUi010-A
