# Supplementary material for: A Human iPSC Line Carrying a de novo Pathogenic FUS Mutation Identified in a Patient With Juvenile ALS Differentiated Into Motor Neurons With Pathological Characteristics
Source: Front Cell Neurosci. 2020 Sep 8;14:273. doi: 10.3389/fncel.2020.00273 (PMC7507938; doi:10.3389/fncel.2020.00273)
Supplement: Supplementary file 1 [file Table_1.DOCX]

Supplementary Material

# Supplementary Tables

Supplementary Table 1. Reagent details

| Antibodies used for immunocytochemistry/flow cytometry | | | |
| --- | --- | --- | --- |
|  | Antibody | Dilution | Company Cat # and RRID |
| Pluripotency markers | Mouse anti-OCT4 | 1:10 | BD Cat# 560186; RRID: AB_1645331 |
|  | Mouse anti-TRA-1-60 | 1:20 | BD Cat# 560850; RRID: AB_165983 |
|  | Mouse anti-SSEA4 | 1:10 | BD Cat# 560128; RRID: AB_1645533 |
|  | Mouse anti-OCT4 | 1:200 | BD Cat# 561555; RRID: AB_10715577 |
| Differentiation markers | Rabbit anti Brachyury | 1:1,600 | Cell Signaling Cat# 81694; RRID: AB_2799983 |
|  | Mouse anti-AFP | 1:20 | BD Cat# 561664; RRID: AB_10895587 |
|  | Mouse anti-Pax6 | 1:20 | BD Cat# 563016; RRID: AB_2737952 |
|  | Anti-OLIG2 | 1:500 | R&D Cat# AF2418 |
|  | Anti-MAP2 | 1:500 | Sigma Cat# M2320 |
| FUS | Anti-FUS | 1:100 | Abcam Cat# ab124923 |
| Secondary antibodies | AF546 donkey anti-mouse IgG | 1:800 | Invitrogen Cat# A10036; RRID: AB_2534012 |
|  | AF488 donkey anti-rabbit IgG | 1:800 | Invitrogen Cat# A21206; RRID: AB_2535792 |

# Supplementary Figures

**Supplementary Figure 1.** Sendai virus residue detection, mycoplasma test and short tandem repeat (STR) Analysis

(**A**) The hiPSCs tested negative for Sendai virus remains. (**B**) The hiPSCs were negative at 280bp suggested no mycoplasma infection. (**C**) Detection sites and results of Short Tandem Repeat (STR) verification of PBMCs from this patient and hiPSCs.
