# Supplementary figures and images for: A Human iPSC Line Carrying a de novo Pathogenic FUS Mutation Identified in a Patient With Juvenile ALS Differentiated Into Motor Neurons With Pathological Characteristics
Source: Front Cell Neurosci. 2020 Sep 8;14:273. doi: 10.3389/fncel.2020.00273 (PMC7507938; doi:10.3389/fncel.2020.00273)

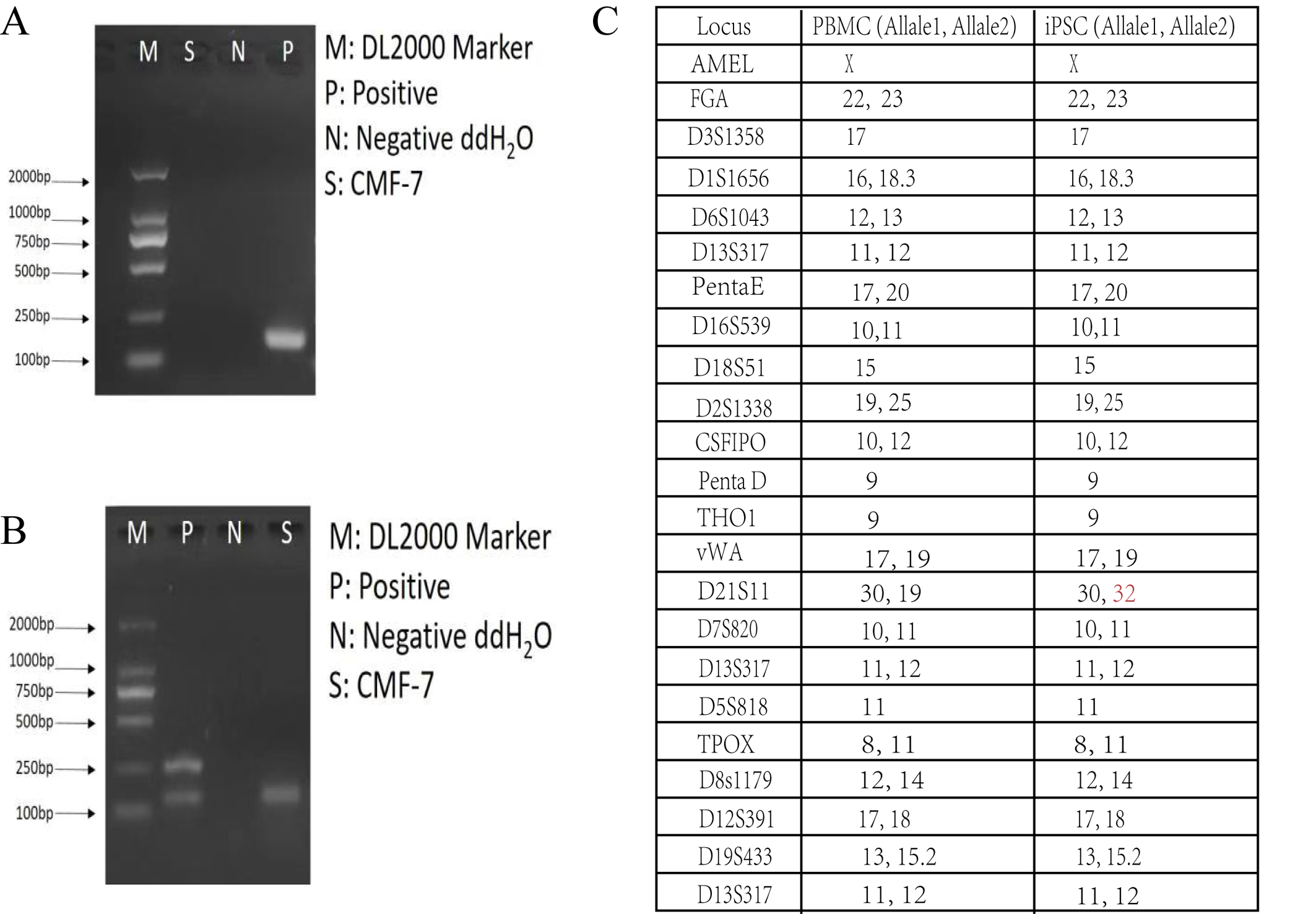

Supplement: Supplementary file 2 [file Image_1.TIF]
